# Adipo/cytokines in atherosclerotic secretomes: increased visfatin levels in unstable carotid plaque

**DOI:** 10.1186/s12872-016-0320-5

**Published:** 2016-07-08

**Authors:** Teresa Auguet, Gemma Aragonès, Esther Guiu-Jurado, Alba Berlanga, Marta Curriu, Salomé Martinez, Ajla Alibalic, Carmen Aguilar, María-Luisa Camara, Esteban Hernández, Xavier Ruyra, Vicente Martín-Paredero, Cristóbal Richart

**Affiliations:** Grup de Recerca GEMMAIR - Medicina Aplicada. Departament de Medicina i Cirurgia, Universitat Rovira i Virgili (URV), Institut Investigació Sanitària Pere Virgili (IISPV), 43007 Tarragona, Spain; Servei Medicina Interna, Hospital Universitari Joan XXIII, 43007 Tarragona, Spain; Servei Anatomia Patològica, Hospital Universitari Joan XXIII, 43007 Tarragona, Spain; Servei Angiologia i Cirurgia Vascular, Hospital Universitari Joan XXIII, 43007 Tarragona, Spain; Servei de Cirurgia Cardíaca, Hospital Germans Trias i Pujol, 08916 Badalona, Spain

**Keywords:** Atheroma plaque, Secretome, Visfatin, Atherosclerosis, Adipo/cytokines

## Abstract

**Background:**

Novel pro-inflammatory and anti-inflammatory derivatives from adipose tissue, known as adipokines, act as metabolic factors. The aim of this study was to analyse the secreted expression of different adipo/cytokines in secretomes of unstable carotid atherosclerotic plaque versus non-atherosclerotic mammary artery.

**Methods:**

We evaluated the secretion levels of adiponectin, visfatin, lipocalin-2, resistin, IL-6 and TNFR2 by ELISA in human secretomes from cultured unstable carotid atherosclerotic plaque (*n* = 18) and non-atherosclerotic mammary artery (*n* = 13). We also measured visfatin serum levels in patients suffering from atherosclerosis and in a serum cohort of healthy subjects (*n* = 16).

**Results:**

We found that visfatin levels were significantly increased in unstable carotid atherosclerotic plaque secretome than in non-atherosclerotic mammary artery secretome. No differences were found with regard the other adipo/cytokines studied. Regarding visfatin circulating levels, there were no differences between unstable carotid atherosclerotic plaque and non-atherosclerotic mammary artery group. However, these visfatin levels were increased in comparison to serum cohort of healthy subjects.

**Conclusions:**

Of all the adipo/cytokines analysed, only visfatin showed increased levels in secretomes of unstable carotid atherosclerotic plaque. Additional human studies are needed to clarify the possible role of visfatin as prognostic factor of unstable carotid atherosclerotic plaque.

## Background

Carotid artery stenosis as a causative factor of ischemic strokes or transient ischemic attacks constitutes a major therapeutic target. Since obesity is considered a risk factor associated to atherosclerosis, a lot of research over recent years has tried to gain greater insights into the link between atherosclerosis and adipose tissue that has been described as an endocrine organ that secretes a wide variety of proteins called adipokines [1–3]. Currently, it is well known that adipokines play a relevant role in the pathophysiology of cardiovascular diseases (CVDs) [4–6]. These molecules can act as enzymes, hormones or growth factors in the modulation of insulin resistance and the metabolism of fats and glucose, and, therefore, have an indirect effect on atherosclerosis [7]. To note, visceral fat accumulation associated with adipokine dysregulation affects on both atherosclerotic plaque development and plaque disruption. When the advanced plaque becomes unstable, rupture can occur and may be provided by the adipokine-induced prothrombotic and inflammatory state [8, 9]. During the last century, the epidemic of obesity and CVDs has lead to intense research into the role of adipokines in obesity and atherosclerosis [6]. However, further research is necessary to elucidate more thoroughly the pathophysiological pathways that underlie the association between adipokines and atherosclerosis, and their potential role as new therapeutic approaches and biomarkers.

Recently, the study of the secretome has emerged as a new strategy for analysing the formation of atherosclerotic plaques in humans [10]. The secretome is the sub-set of proteins released by a cell or tissue under certain conditions and shows a narrower dynamic range of proteins than serum or plasma, which means less complexity. Furthermore, studies on tissue secretome more closely resemble the in vivo situation than cell culture workflows.

The aim of this study was to analyse the presence of several adipo/cytokines with different profiles, pro- and anti-inflammatory: adiponectin, visfatin, lipocalin-2, resistin, IL-6 and TNFR2, and compare their differential expression in the secretome of an unstable carotid atherosclerotic plaque with the secretome in a non-atherosclerotic mammary artery. Moreover, in order to study whether the differences observed in adipo/cytokine levels were only a local effect or if they were also reflected in serum, we measured circulating levels in the group of patients suffering from atherosclerosis and in a serum group of healthy subjects.

## Methods

### Subjects/Samples

The study was approved by the institutional review board “Comitè d’Ètica d’Investigació Clínica, Hospital Universitari de Sant Joan de Reus” (10-04-29/4proj3). All participants gave written informed consent for participation in medical research.

Human unstable carotid atherosclerotic plaques were obtained from patients (men, *n* = 18) who underwent carotid endarterectomy at the Angiology and Vascular Surgery Unit of the Hospital Universitari Joan XXIII (Tarragona, Spain). Patients with cerebrovascular ischemia and internal carotid artery stenosis >75 % were included, diagnosed by colour Doppler assisted duplex investigation and arteriography. The diagnosis of unstable carotid atherosclerotic plaques was made by an experienced pathologist following the American Heart Association (AHA) guidelines [11].

Mammary arteries were used as non-atherosclerotic control arteries. Segments of mammary arteries (men, *n* = 13) were obtained during coronary revascularisation surgery at the Cardiovascular Surgery Department of the Hospital Germans Trias i Pujol (Badalona, Spain). Patients who had an acute illness, acute or chronic inflammatory or infective diseases, or malignant neoplastic disease were excluded.

We also recruited serum cohort of healthy men (*n* = 16), whose medical history included no cardiovascular event. Subjects who had an acute illness, acute or chronic inflammatory or infective diseases, or malignant neoplastic disease were excluded.

All subjects recruited were male. Blood samples were obtained from each individual immediately before surgery and after overnight fasting. Serum was obtained by standard protocols and preserved at −80 °C until use.

### Clinical and biochemical assessments

A complete anthropometric, biochemical, and physical examination was carried out on each patient. Body height and weight were measured with the patient standing in light clothes and shoeless. Body mass index (BMI) was calculated as body weight divided by height squared (kg/m^2^). Laboratory studies included glucose, insulin, glycated haemoglobin (HbA1c), total cholesterol, high-density lipoprotein cholesterol (HDL-C), low-density lipoprotein cholesterol (LDL-C) and triglycerides, all of which were analysed using a conventional automated analyser. Insulin resistance (IR) was estimated using the homeostatic model assessment of IR (HOMA2-IR) [12].

### Arterial tissue culture – obtaining the secretome

Tissue samples were transported from the surgery to the laboratory in phosphate buffered saline (PBS) at room temperature. Immediately upon arrival, the tissue was transferred to a Petri dish and washed with PBS. For mammary arteries, we removed the adventitia before incubation of the intima-media. All samples were then cut into similar-sized pieces about 3–5 mm in length and transferred to a 12-well tissue culture plate containing 2 ml/well of protein-free Roswell Park Memorial Institute medium (RPMI) (RPMI-1640, Gibco, Invitrogen, N.Y, USA) supplemented with penicillin (100 U/ml), streptomycin (100 μg/ml) and 50 mM HEPES. These procedures were all carried out under a laminar flow hood using sterile equipment. After 24 h of incubation at 37 °C and 5 % of CO_2_, the media containing the secreted proteins, the so-called secretome, were collected, aliquoted and stored at −80 °C until used for analysis.

Additionally, a section of each atherosclerotic plaque was placed in phormol 10 % and further studied by an experienced pathologist from the Hospital Universitari Joan XXIII (Tarragona) following the AHA guidelines [11].

### Measurements of adipo/cytokines levels

Defrosted secretome samples were centrifuged at 1200 rpm and 4 °C for 15 min. Then, they were analysed by enzyme-linked immunosorbent assays (ELISA) following the manufacturer’s instructions. Adiponectin (EMD Millipore, St. Charles, MI, USA), visfatin (AdipoGen, San Diego, CA, USA), lipocalin-2 (R&D Systems Inc, Minneapolis, USA), resistin (Biovendor, Modrice, Czech Republic), IL-6 (R&D Systems Inc, Minneapolis, USA) and TNFR2 (BioSource Europe, Nivelles, Belgium) were determined in secretome samples. Only visfatin was determined in both secretome and serum samples. The adiponectin assay sensitivity was 0.2 ng/ml, and intra-assay and inter-assay coefficients of variation (CV) were 3.4 and 5.7, respectively. The visfatin assay sensitivity was 30 pg/ml, and intra-assay and inter-assay CV were 5.63 and 5.92, respectively. The lipocalin-2 assay sensitivity was 0.012 ng/ml, and intra-assay and inter-assay CV were 3.7 and 6.5, respectively. The resistin assay sensitivity was 0.012 ng/ml, and intra-assay and inter-assay CV were 5.9 and 7.6, respectively. The IL-6 assay sensitivity was 0.039 pg/mL, and intra-assay and inter-assay CV were 7.4 and 7.8, respectively. Finally, sTNF-RII assay sensitivity was 0.1 ng/ml, and intra-assay and inter-assay CV were 4.9 and 7.9, respectively. In order to normalize adipo/cytokine measurements, total protein concentration was assessed using the Pierce BCA protein assay kit (Thermo Scientific, Waltham, MA, USA) following the manufacturer’s instructions.

### Statistical analysis

All the values reported are expressed as mean ± standard deviation (SD) and were analysed using the Windows SPSS/PC+ statistical package (version 22.0; SPSS, Chicago, IL, USA). Differences between groups were calculated using Student’s t test or one-way ANOVA analysis. The strength of association between variables was calculated using Pearson’s method for parametric variables and the Spearman Rho correlation test for non-parametric contrasts. *P* values <0.05 were considered to be statistically significant.

## Results

### Characteristics of the population studied

The general characteristics and biochemical measurements of the population studied are shown in Table 1. Subjects were classified according to the samples obtained: serum group of healthy subjects (*n* = 16), non-atherosclerotic mammary artery samples from patients undergoing coronary artery bypass (*n* = 13) and unstable carotid atherosclerotic plaque samples from patients undergoing endarterectomy (*n* = 18). The three groups studied had similar BMIs and they were all men. Anthropometrical and biochemical parameters showed no significant differences between non-atherosclerotic mammary artery and unstable carotid atherosclerotic plaque groups. As expected, carotid atherosclerotic plaque and mammary artery patients showed significant lower lipid profile because these subjects were taking lipid-lowering drugs. Table 1 also shows that the levels of glucose and HbA1c were significantly higher in the carotid atherosclerotic plaque and mammary artery group than in serum group of healthy subjects.

**Table 1:** Anthropometric measurements and metabolic analysis of the population studied

	Serum group of healthy subjects	Coronary patients with non-atherosclerotic mammary artery	Unstable carotid atherosclerotic plaque group
	(*n* = 16)	(*n* = 13)	(*n* = 18)
	Mean ± SD	Mean ± SD	Mean ± SD
Age (years)	52.47 ± 13.25	65.08 ± 10.48	69.17 ± 7.44^b^
BMI (kg/m^2^)	32.19 ± 11.76	29.39 ± 3.36	27.74 ± 3.13
Glucose (mg/dl)	91.31 ± 14.24	129.19 ± 55.44^a^	123.56 ± 45.37^b^
HbA1c (%)	4.97 ± 0.39	6.81 ± 1.39^a^	6.29 ± 1.07^b^
Insulin (mUI/L)	12.76 ± 16.19	11.76 ± 7.15	7.21 ± 4.93
HOMA2-IR	1.62 ± 1.95	1.59 ± 0.97	1.01 ± 0.67
Triglycerides (mg/dL)	115.02 ± 71.38	110.33 ± 27.84	103.00 ± 40.61
Cholesterol (mg/dl)	192.33 ± 37.81	128.34 ± 23.92^a^	118.81 ± 34.54^b^
HDL-C (mg/dL)	49.13 ± 10.35	23.71 ± 4.64^a^	28.50 ± 6.98^b^
LDL-C (mg/dL)	120.17 ± 39.06	78.56 ± 19.48^a^	69.78 ± 26.21^b^

Subjects were classified according to the samples obtained: serum group of healthy subjects (*n* = 16), non-atherosclerotic mammary artery samples from patients undergoing coronary artery bypass (*n* = 13) and unstable carotid atherosclerotic plaque samples from patients undergoing endarterectomy (*n* = 18). *BMI* body mass index, *HbA1c* glycosylated haemoglobin, *HOMA2-IR* homeostatic model assessment 2- insulin resistance, *HDL-C* high density lipoprotein, *LDL-C* low density lipoprotein. Data are expressed as mean ± SD. *p* <0.05 are considered statistically significant. ^a^refer to the statistically significant differences between coronary patients with non-atherosclerotic mammary artery and serum group of healthy subjects. ^b^refer to the statistically significant differences between unstable carotid plaque and serum group of healthy subjects. HOMA-2 is calculated using the HOMA Calculator version 2.2.2 (http://www.dtu.ox.ac.uk)

### Adipo/cytokine levels in the secretome

To study the local role of adipo/cytokines in atherosclerosis, we evaluated the presence of adiponectin, visfatin, lipocalin-2, resistin, IL-6 and TNFR2 in secretomes of the unstable carotid atherosclerotic plaque and non-atherosclerotic mammary artery tissue cultures (Table 2). Of all the molecules analysed, visfatin was the only adipo/cytokine that was differently expressed in secretome samples. Specifically, visfatin levels were significantly higher in the unstable carotid atherosclerotic plaque than in non-atherosclerotic mammary artery secretomes (Table 2, *p* = 0.021). Conversely, the levels of adiponectin and IL-6 showed no significant differences between the two secretome groups analysed. Finally, the levels of lipocalin-2, resistin and TNFR2 were almost undetectable in the secretome samples. No significant correlations between adipo/cytokines were found.

**Table 2:** Adipo/cytokine levels in secretome samples

	Unstable carotid atherosclerotic plaque group	Coronary patients with non-atherosclerotic mammary artery
	(*n* = 18)	(*n* = 13)
	Mean ± SD	Mean ± SD
Visfatin (ng/μg total protein)	0.100 ± 0.017	0.046 ± 0.012^a^
Adiponectin (μg/μg total protein)	0.311 ± 0.039	0.369 ± 0.096
IL-6 (pg/μg total protein)	0.048 ± 0.012	0.039 ± 0.008
Lipocalin-2 (ng/μg total protein)	0.009 ± 0.002	0.008 ± 0.001
Resistin (ng/μg total protein)	0.001 ± 0.001	0.001 ± 0.001
TNFR2 (ng/μg total protein)	0.007 ± 0.002	0.005 ± 0.001

*IL-6* interleukin 6, *TNFR2* tumor necrosis factor receptor 2. Data are expressed as mean ± SD. *p* <0.05 are considered statistically significant. ^a^refer to the statistically significant differences between unstable carotid atherosclerotic plaque and non-atherosclerotic mammary artery group

### Circulating Visfatin and adipocytokines levels in serum

**Fig. 1:**
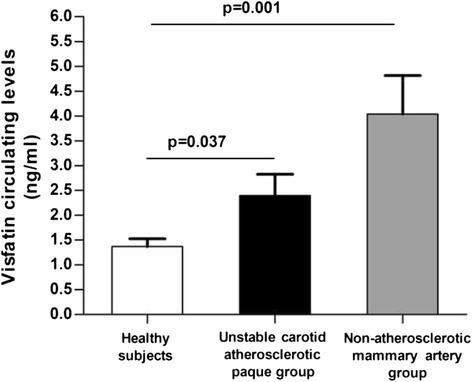
Visfatin serum levels in different groups: unstable carotid atherosclerotic plaque group (*n* = 18), coronary patients with non-atherosclerotic mammary artery (*n* = 13) and serum cohort of healthy subjects (*n* = 16). *p* <0.05 were considered statistically significant

As only differences in situ visfatin levels were observed and in order to study whether these differences were only a local effect or if they were also reflected in serum, we measured visfatin circulating levels in the group of patients suffering from atherosclerosis and in a serum group of healthy subjects (*n* = 16). Fig. 1 shows that there were no differences between unstable carotid atherosclerotic plaque and non-atherosclerotic mammary artery group. However, visfatin serum concentration was higher in both unstable carotid atherosclerotic plaque and non-atherosclerotic mammary artery groups than in the serum cohort of healthy subjects (*p* = 0.037 and p = 0.001; respectively). This difference remained significant after adjusting for age, BMI and glucose metabolism.

Then, we analysed the circulating levels of two adipo/cytokines with different profile, pro- and anti-inflammatory (IL-6 and adiponectin, respectively). We found that adiponectin circulating levels were significantly higher in the serum group of healthy subjects (29.20 ± 8.42) than unstable carotid atherosclerotic plaque group (11.23 ± 1.69, *p* = 0.025) and non-atherosclerotic mammary artery patients (9.26 ± 2.35, *p* = 0.031). However, we observed no differences in the circulating levels of IL-6 between groups. No significant correlations between these adipo/cytokines and visfatin were found.

## Discussion

To date, the knowledge of the local action of the adipo/cytokines expressed in secretomes of atherosclerotic plaques is under development. In fact, most secretome studies have been carried out using proteomic techniques [10, 13]. The aim of this study was to analyse the presence of several adipo/cytokines with different profiles, pro- and anti-inflammatory in the secretome of an unstable carotid atherosclerotic plaque with the secretome in a non-atherosclerotic mammary artery. The main finding was that visfatin levels were significantly higher in the unstable carotid atherosclerotic plaque than in non-atherosclerotic mammary artery secretomes, suggesting a possible link between visfatin and unstable carotid atherosclerotic plaque.

Visfatin is a ubiquitous adipokine that is produced in adipose tissue, bone marrow, skeletal muscle, and liver with a physiological role not completely understood [14–16]. In the context of metabolic diseases, most studies have focused on increased circulating levels and adipose tissue expression of visfatin [17, 18]. Also, it was initially proposed as a clinical marker of atherosclerosis, endothelial dysfunction and vascular damage [19]. Also, visfatin is an active player promoting vascular inflammation, atherosclerosis development and progression, and plaque destabilization [19–21]. As far as the local effect of visfatin on atherosclerotic lesions is concerned, other authors studying the atheroma plaque directly have reported similar results to ours [22–24]. One of the studies that has most similarities with ours has reported that visfatin should be regarded as an inflammatory mediator, localized to foam cell macrophages within unstable atherosclerotic lesions, which potentially plays a role in plaque destabilization [22]. Moreover, Zhou et al. have reported that visfatin induces cholesterol accumulation in macrophages and accelerates the process of atherosclerosis [23]. Apart from the pro-inflammatory effect of visfatin on atherosclerosis, other possible direct mechanisms have been reported: promotion of smooth muscle cell proliferation, alteration of the expression and the activity of matrix metalloproteinases, greater atherosclerotic plaque vulnerability and impairment of endothelial vasodilatory responses [25–28]. The mechanism underlying elevated levels of visfatin in secretomes from unstable atherosclerotic plaques are not known nowadays. However, Dahl et al. have reported enhanced visfatin expression in symptomatic atherosclerotic plaques and also that visfatin had a combined ability of increasing TNF-α as well as to respond with increased expression on TNF-α stimulation. Therefore, this bidirectional interaction between TNF-α and visfatin could represent a pathogenic loop on unstable atherosclerotic lesions [22]. Another study has further demonstrated that the regulation of visfatin in macrophages is related to pro-atherogenic stimuli, including hypoxia, TNF-α and ox-LDL [29].

Although some authors have identified that visfatin is a potential inflammatory mediator in plaque destabilization [22], in our study we only included patients with cerebrovascular ischemia and unstable carotid atherosclerotic plaque. Therefore, we could not compare visfatin levels between stable and unstable carotid plaque secretomes. Although the biological mechanisms involving visfatin in the pathogenesis of atherosclerosis are not well-established, visfatin seems to be an active factor in the development and progression of atherosclerosis through its effects on cytokine and chemokine secretion, macrophage survival, leukocyte recruitment by endothelial cells, vascular smooth muscle inflammation and plaque destabilization [19, 23].

Regarding circulating levels, we found higher visfatin serum concentrations in patients suffering carotid atherosclerosis and coronary patients with non-atherosclerosis mammary artery who underwent coronary revascularisation surgery. In our study, mammary arteries have been used as control arteries, since previous studies have shown its lower incidence of atherosclerosis [30, 31]. However, it is important to remark that although mammary artery patients have non-diseased arterial secretome, they have atherosclerotic coronary disease. Likewise, in recent years, several studies have established positive associations between enhanced circulating visfatin levels and atherogenic inflammatory diseases, which suggest a possible role of visfatin in the atherosclerosis pathogenesis [19, 32]. Specifically, visfatin was associated with infarct-related artery occlusion, and also an association with coronary artery disease was found [33, 34]. On the other hand, some authors claim that high visfatin levels, instead of depicting changes in the atherosclerotic process are more likely to reflect changes in systemic inflammation in patients with cardiovascular disease [19]. Although our local and systemic results reinforce the first hypothesis, additional human studies are needed if these data are to be clarified.

Regarding the other adipo/cytokines, levels of lipocalin-2, resistin and TNFR2 were almost undetectable in the secretome samples. In addition, the levels of adiponectin and IL-6 showed no differences between the two secretome groups analysed. Several studies have described a protective role of adiponectin in cardiovascular diseases [35]. Although we did not find differences between secretome groups, we found higher serum levels of adiponectin in control individuals than in both unstable carotid atherosclerosis and non-atherosclerotic mammary artery patients. Further studies are needed to assess whether adiponectin can have a direct effect in situ by inhibiting the formation of an atherosclerotic plaque. Although IL-6 has been regarded as a pro-inflammatory cytokine that is classically associated with endothelial dysfunction and atherosclerosis [36], we found no differences in secretome or circulating levels between the group of patients suffering from atherosclerosis and the mammary artery. The reason for these discrepancies could be the dissimilarities of the studied populations.

Our results require the following observations. First, we have used mammary arteries as control arteries, since previous studies have shown a lower incidence of atherosclerosis. However, non-atherosclerotic carotid arteries would be the best choice but, unfortunately, are not available. Second, this study was cross-sectional, so it allowed us to detect correlations but not to formulate predictions. Future prospective studies are necessary to elucidate more thoroughly the association between some molecules such as visfatin and atherosclerosis, and also their potential role as new therapeutic approaches and biomarkers of unstable vs. stable plaques. As our study was conducted only including unstable plaques, we could only suggest doing further in order to confirm this hypothesis.

## Conclusions

Of all the adipo/cytokines analysed in secretomes, visfatin was the only adipo/cytokine that was higher in unstable carotid artery plaque than in non-atherosclerotic mammary artery secretomes. Regarding visfatin serum levels, there were no differences between unstable carotid atherosclerotic plaque and non-atherosclerotic mammary artery groups. However, these visfatin circulating levels were increased in comparison to serum cohort of healthy subjects. Prospective studies are needed to confirm whether visfatin could play a role as prognostic factor in the stability of atherosclerotic plaque.

## Abbreviations

BMI, body mass index; HbA1c, glycosylated hemoglobin; HDL-C, high density lipoprotein; HOMA2-IR, homeostatic model assessment method insulin resistance; IL-6, interleukin 6; LDL-C, low density lipoprotein; PBS, phosphate buffered saline; RPMI, protein-free Roswell Park Memorial Institute medium; TNFR2, tumor necrosis factor receptor 2
